# Why Quantification Matters: Characterization of Phenotypes at the *Drosophila* Larval Neuromuscular Junction

**DOI:** 10.3791/53821

**Published:** 2016-05-12

**Authors:** Mario Sanhueza, Anisha Kubasik-Thayil, Giuseppa Pennetta

**Affiliations:** ^1^Euan MacDonald Centre for Motor Neurone Disease Research, University of Edinburgh; ^2^School of Biomedical Sciences, University of Edinburgh

**Keywords:** Neuroscience, Issue 111, neuromuscular junction, nucleus, nuclear morphology, nuclear position, synaptic structure, *Drosophila*, phenotypic quantification

## Abstract

Most studies on morphogenesis rely on qualitative descriptions of how anatomical traits are affected by the disruption of specific genes and genetic pathways. Quantitative descriptions are rarely performed, although genetic manipulations produce a range of phenotypic effects and variations are observed even among individuals within control groups. Emerging evidence shows that morphology, size and location of organelles play a previously underappreciated, yet fundamental role in cell function and survival. Here we provide step-by-step instructions for performing quantitative analyses of phenotypes at the *Drosophila* larval neuromuscular junction (NMJ). We use several reliable immuno-histochemical markers combined with bio-imaging techniques and morphometric analyses to examine the effects of genetic mutations on specific cellular processes. In particular, we focus on the quantitative analysis of phenotypes affecting morphology, size and position of nuclei within the striated muscles of *Drosophila *larvae. The *Drosophila* larval NMJ is a valuable experimental model to investigate the molecular mechanisms underlying the structure and the function of the neuromuscular system, both in health and disease. However, the methodologies we describe here can be extended to other systems as well.

**Figure:**
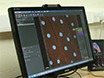


## Introduction

Qualitative analysis restricts the focus of most experimental studies to the examination of genetic manipulations leading to large phenotypic effects or to phenotypes that are not amenable to quantification, *e.g.*, absence/presence. Quantification of phenotypes is not usually performed and variations present within members of a phenotypic group are not taken into consideration. Additionally, without mathematical descriptions of morphologies, it may be difficult to determine whether fine scale phenotypic changes are the result of genetically induced alterations or whether observed changes are simply due to random fluctuations. 

We propose that for an accurate and unbiased analysis of phenotypic defects due to gene disruption, quantitative methodologies should accompany more traditional qualitative approaches. Quantitative evaluation of phenotypes is particularly beneficial for structures such as synapses that present a high degree of variability due to their intrinsic morphological and functional plasticity. We have selected examples of quantitative analyses applied to areas of investigation with which we are most familiar, namely the *Drosophila *larval neuromuscular junctions (NMJs). However, concepts and principles apply equally well to other experimental systems. 

The larval NMJ is an excellent model system to study synaptic development and function because of the highly stereotyped nature of its structure. Each hemi-segment of the larval neuromuscular system contains 32 identifiable motor neurons forming synaptic contacts with their postsynaptic target muscle. Every hemi-segment also contains a fixed number of muscles visible as multinucleated fibers attached to the internal surface of the cuticle^1^. Another advantage of using the larval neuromuscular system is the power and versatility of *Drosophila* genetics that easily allows the generation of a number of mutant alleles and the possibility to modify gene expression in a time- and tissue-restricted manner. Finally, 75% of the human genes causing a disease have an evolutionarily conserved orthologue in *Drosophila*^2^. Indeed, entire genetic pathways are conserved between flies and humans. Because of this, the *Drosophila* larval neuromuscular system is a very popular experimental model to elucidate the molecular mechanisms underlying a number of human diseases including amyotrophic lateral sclerosis (ALS)^1^. 

Here we show that the availability of several reliable immuno-histochemical markers, combined with bio-imaging techniques and accurate morphometric analyses can describe anatomical traits that are likely to play an important functional role^3,4,5^. Among the cellular processes that are amenable to quantitative analyses, we focus on changes in shape, size and position of intracellular structures such as the nuclei. All these are processes that we know very little about.

The challenge for molecular geneticists in the coming decades will be to extend our current knowledge by analyzing the effect of genetic mutations that produce very subtle phenotypic defects. Quantitative methodologies that allow researchers to meticulously explore the effects of genetic mutations can provide a more comprehensive understanding of how genotypes relate to phenotypes, especially for poorly understood cellular processes. 

## Protocol

### 1. Experimental Preparation

Note: Dissections and immuno-histochemistry procedures in sections 2 and 3 are performed according to references^3-6^, but with modifications.

1. Prepare 1x Phosphate-buffered Saline (PBS) and PBS containing 0.1% Triton-X 100 (PBT). Keep them on ice.
2. Prepare Bouin's fixative (15 Picric Acid:10 Formaldehyde:1 Glacial Acetic Acid). Make this reagent fresh.
3. Select clean stainless steel minutien pins and fine forceps.
4. Prepare dissection plates containing a Sylgard disc in a 5 cm Petri dish.

### 2. Dissection of Third-instar Larval NMJs

1. Pick wandering third-instar larvae from a vial or a bottle with a fine brush and place them into a 2 cm Petri dish containing 4 °C PBS to wash the residual food away.
2. Place one larva on the top of the sylgard surface of the dissection plate and make sure that it is positioned with its dorsal side up so that the two longitudinal tracheal tubes are visible on the top.
3. Using the forceps to hold the pin, pin the larva down at its anterior end, right under the mouth hook. Stretch the larva out as much as possible and pin its posterior end down.
4. Add enough PBS saline to reach the walls of the plate and completely immerse the larva.
5. Repeat the procedure from steps 2.1 to 2.3 for other larvae of the same genotype. A single 5 cm Petri dish dissection plate can easily accommodate up to 8 larvae.
6. Using the micro-dissection scissors, slightly lift the dorsal cuticle and make a small horizontal incision at the posterior end near the pin.
7. Insert scissors into the incision, cut the larva all the way to the anterior end along the midline between the two longitudinal tracts of the trachea. Make sure that the midline cuts are superficial enough to just pass through the cuticle and to avoid cutting through the muscles on the ventral side.
8. At each end, cut two notches on both the left and the right sides.
9. Open the fillet by placing two pins on both sides of the anterior incision. Repeat the same with the posterior end. When placing the pins, make sure to spread the body wall apart.
10. Clean out the internal organs using forceps and PBS saline. Leave the central nervous system intact. Gently stretch the larva with corner pins until it is completely stretched but make sure that the muscles are not torn during this process.
11. Repeat the same dissection procedure for the other larvae on the same dissection plate.
12. Wash with PBS saline three times to remove all the internal organs.
13. Replace the PBS with Bouin's fixative and leave at room temperature for 10 min.
14. Wash several times with PBT.
15. Remove the pins carefully and transfer all the preparations, which are now fairly rigid, into a 1.5 ml micro-centrifuge tube for immuno-staining.

### 3. Immuno-histochemical Staining of *Drosophila *NMJs with Antibodies Specific for Muscles and Myonuclei

1. Quickly rinse the larval fillets in PBT.
2. Block the preparations by incubating with 10% normal goat serum (NGS) in PBT for 2 hr under constant agitation.
3. Incubate a set of dissected NMJs in PBT containing 5% of NGS and a rabbit anti-lamin antibody (at a concentration of 1:500) and with a guinea pig anti-DVAP antibody (at a concentration of 1:500) for 2 hr at room temperature or at 4 ºC overnight. The anti-DVAP antibody stains the striated muscles while the anti-lamin staining detects the contour of myonuclei.
4. Wash once quickly in PBT to remove the antibodies in excess. Wash in PBT for 2 hr by changing the PBT buffer every 15 min.
5. Incubate the samples in PBT containing 5% NGS and fluorescent-labelled secondary antibodies at 1:500 dilution for 2 hr at room temperature. The same samples can be subjected to staining with multiple antibodies at the same time if secondary antibodies conjugated with different chromophores are used.
6. Remove secondary antibodies and wash in PBT for 2 hr by changing the PBT buffer every 15 min.
7. To stain the interior of the myonuclei wash the samples three time with PBS and proceed as follows:
  1. Add the nuclear marker TO-PRO-3 at a dilution of 1:1,000 in PBS and incubate for 20 min under constant agitation. This marker is used in this study but any other commercially available nuclear marker can be used as well.
  2. Wash quickly three times in PBS before mounting.

### 4. Mounting Samples on the Slides

1. Pick the samples up with forceps from the 1.5 ml micro-centrifuge tube and lay them down on a processing slide.
2. By using micro-dissection scissors, cut the head and the tail of the fillets and keep their internal surface up.
3. Prepare the mounting slide by wrapping around three strips of cellulose tape on each side of a clean slide at a distance of about 1 cm from each other. Once the coverslip is positioned on top of the two strips, a gap will be generated that will avoid flattening of the samples. This is crucial if three-dimensional volume renderings of structures are to be made.
4. Put a small drop of about 20 µl of the mounting medium in the middle of the mounting slide between the three cellulose tape strips.
5. After spreading the mounting medium with clean forceps, drag the dissected larvae to the mounting slide into the mounting medium, keeping the internal surface up. Try to mount them in rows of four or five.
6. Gently drop a cover slip on top of the mounting slide and make sure that no air bubbles are generated. Seal the slide with transparent nail varnish. Let the samples dry for at least 10 min before imaging.

### 5. Confocal Settings for Imaging

Note: Images presented in this study are taken using a Nikon A1R confocal unit integrated onto a Ti:E inverted microscope. However, any confocal microscope with a minimum of 3 laser units available in the wavelength regions of 488 nm, 561 nm and 642 nm and a 3 channel detection system is suitable for this purpose.

1. Turn ON the lasers, detector unit, mercury bulb, stage controller, microscope and the PC. Start the control software and secure the slide on the stage holder.
2. To make imaging faster, select and mark all the regions of interest (ROI) on the sample by using a 20X objective.
3. Carefully swing the nosepiece to the 60X higher magnification objective lens (60X Plan Apo VC/ NA 1.4 OIL).
4. Place a drop of immersion oil onto the objective lens and select one of the marked ROI from XYZ overview window on the computer.
5. Start imaging by using the following optical settings: Select the first Dichroic Mirror: 405/488/561/640. Select 488 nm laser with emission filter 525/50 nm in channel 1, 561 nm laser with 595/50 emission filter in channel 2 and 642 nm laser with long-pass filter 650 nm in channel 3.
6. Use the following scan settings before starting image acquisition: Select Galvano scanner; Scan direction: one way; Scan speed: 0.5 frames per sec.
7. Select Channel series to avoid channel bleed-through.
8. Adjust the pin-hole size to 1 airy unit. Scan and adjust the laser power, detection gain and offset appropriately for each channel to avoid pixel saturation and background level.
9. Acquire z-stacks by using voxel size 0.2 x 0.2 x 0.5 µm^3^ for all the ROIs and the slide preparations. If the images will be subjected to deconvolution then set the voxel size to 0.06 x 0.06 x 0.15 µm^3^.
10. Save images in .nd2 file format or .ics format.

### 6. Calculation of the Distance Between Nuclei within Striated Muscles by the Method of the Nearest Neighbor Analysis

1. For this analysis, use confocal images displaying larval body-wall muscles stained with DVAP antibodies to visualize the muscles and with lamin antibodies and a nuclear marker to highlight the nuclei.
2. To estimate the nearest distance between nuclei, use Measurement Points within the MeasurementPro module of the image analysis software (*e.g.*, Imaris). Other similar software applications for image analysis can be used for the same purposes.
3. Open the confocal images. To initiate, double click on the software icon. Drag and drop the confocal z-stack images into the Arena.
4. Double click on the images to automatically open them in the Surpass View, under the menu tool bar icon  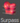. The Surpass View has three main workspace panels: View Area, Object List and Object Properties Area.
5. Create a volume rendered three channel image by clicking on the menu icon 3D View 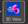.
6. Click on the Add New Measurement Points icon 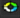 from the****Objects toolbar and follow the creation wizard that appears in Object Properties Area.
7. From the creation wizard select the Edit tab first and then Specific Channel. Select either the nuclear marker or the lamin channel to highlight the nuclei.
8. Set the pointer to the Select mode by pressing the Esc tab on the keyboard.
9. Adjust the size of the 3D cursor box with the mouse wheel to contain a given nucleus in the image. Add a measurement point by holding the Shift key and left mouse click on the same nucleus.
10. Add the second point on a nearby nucleus on the same muscle by repeating the previous steps. A line is automatically drawn between the two points and the measured distance between the two nuclei is displayed. The distance between the two nuclei is now recorded as a statistical variable in the Object Properties Area under the tab Statistics >Detailed>Distance data.
11. Repeat the procedure from steps 6.6 to 6.10 for all nuclei surrounding a given nucleus.
12. From Statistics > Detailed> Distance data displaying all collected measurement points, select the shortest distance.
13. By clicking on the Export Statistics on Tab Display to File 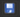 available under the Object Properties Area, the data will be saved on a spreadsheet.
14. Repeat the same procedure for the other surrounding nuclei and for a selected number of muscle fibers per genotype.
15. Using the data exported to a spreadsheet file, calculate the average shortest distance (D_ave_) for all the M number of muscles using the following equation: 
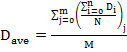
 Di is the shortest distance to the neighboring nucleus for a given nucleus i where i varies from 0 to N and N is the number of nuclei analyzed per muscle. The summation of values from j = 0 to j = m indicates the M number of analyzed muscles.
16. Alternatively, use****Spots Creation wizard and Spots to Spots Closest Distance to estimate the average distance to the nearest neighboring nucleus. For this, a Matlab extension module is needed.
  1. Double click on one specific image on the Arena and a 3D-volume image will be displayed in the View Area. Click on the Object Creation icon and Add New Spots 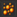 from the Objects toolbar.
  2. From the creation wizard in the Object Properties Area, click on the option Skip Automatic Creation, Edit Manually.
  3. Select either the lamin or nuclear marker channel to display the nuclei.
  4. Shift and click left mouse button on all the nuclei of a specific muscle in the image. A spot on every nucleus will appear.
  5. Select Spots to Spots Closest Distance listed under the Tools tab in the Object Properties Area. Select Spots Statistics under the Result mode and a Matlab window appears.
  6. Under the Statistics tab, select Detailed and then Specific Values. Click on the Distmin and the values of the minimum distances for every nucleus will appear.
  7. Export the data to a spreadsheet file and calculate the average of these distances per muscle. Repeat the procedure for all muscles of a given genotype.

### 7. Determining the Shape of Nuclei within the Body-wall Muscles of *Drosophila* Larvae

1. For this analysis, use confocal images of body-wall muscles stained with lamin and a nuclear marker to visualize the nuclei.
2. To evaluate the shape of myonuclei, measure sphericity (defined as the ratio of the surface area of a sphere with the same volume as the given nucleus, to the surface area of the nucleus) or ellipticity (distinguishes between oblate/prolate ellipsoids and spheroids).
3. Open the image as previously described. Click on the Objects toolbar icon Add New Surfaces 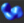.
4. In the creation wizard that appears in the Object Properties Area, select the nuclear marker staining as the Source Channel to display the nuclei.
5. Set the option Absolute Intensity as the threshold. Make sure that most of the nuclei show a smooth and not overloaded rendering by changing the value on the threshold curve. At the same time, avoid the presence of holes or incomplete mask to any nucleus using the same curve.
6. Use the****Filter tool to exclude any noise in the surface rendering. Under the Edit tab of the newly created surface layer, Split or Merge the nuclei surfaces that are incorrectly rendered.
7. Export to a spreadsheet file the ellipticity and sphericity values of the surface rendered nuclei that are available under the Statistics tab.
8. Alternatively, use ImageJ or Fiji software to measure the circularity of nuclei where the Circularity (C) is defined as C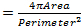 . A value of one represents a perfect circle while a value approaching zero indicates an increasingly elongated shape.
9. Create maximum intensity projections of the z stacks from menu Images>Stacks>Z Project. Set projection type to Max Intensity.
10. Split the channels and Select the nuclear marker channel.
11. From the main menu, select Image>Adjust>Threshold.
12. Segment the nuclei by adjusting the intensity threshold. If the nearby nuclei are segmented as a single unit, click on Process>Binary>Watershed tool to disconnect the nuclei.
13. From the main menu, select Edit>Selection>Create selection.
14. Add all the selected ROIs to the ROI Manager by clicking on menu Analyze>Tools>ROI Manager. In the ROI Manager window click on Add. Within the same window select More >Split.
15. Select Shape Descriptors in Analyze>Set Measurements. In the ROI Manager window click on the tab****Measure. This will display a list of circularity values of all the selected nuclei.
16. In addition, to measure the nuclear volume, follow surface creation wizard using the nuclear marker staining channel as described in subsections 7.3-7.7.

### 8. 3D Volume Renderings of Selected Nuclei within the *Drosophila* Larval Body-wall Muscles to Evaluate the Intranuclear Localization of a Specific Protein

1. Use confocal images reporting body wall muscles stained with a nuclear marker and with antibodies specific to lamin and DVAP.
2. Open the images by initiating the software and select the specific nucleus to be analyzed. This can be done by selecting the main menu item Edit>Crop 3D.
3. Follow surface creation wizard using lamin channel as described in subsection 7.3-7.7.
4. Once the surface is created, click on Edit in the Objects Properties Area and then select Mask All to isolate the signal inside the nucleus. This creates a new window.
5. Select DVAP signal from the Select Channel drop-down menu. Select the DVAP immuno-reactivity signal inside the nucleus by clicking on the option Set Voxels Outside The Surface to zero. A new masked channel is created and is available in the display adjustment window for selection.
6. To visualize the presence of signal inside the nucleus create a contour plane by clicking on the icon Add New Clipping Plane 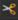 from the Objects toolbar.      
7. Interactively adjust the angle of the clipping plane and its position to visualize the distribution of the signal inside the nucleus.

## Representative Results

ALS is a degenerative disease specifically affecting motor neurons leading to a progressive and fatal paralysis of striated muscles^7^. Missense mutations in the human VAMP-Associated Protein B (hVAPB) cause a range of motor neuron diseases including ALS type 8^8-12^. A missense mutation (V234I) in the hVAPB gene has been recently identified in one case of typical ALS in humans^13^. To assess its pathogenic potential, we generated transgenic flies expressing the hVAPB *Drosophila* orthologue DVAP carrying the disease-causing mutation (DVAP-V260I). The expression of this transgene was targeted to the muscles using the UAS/GAL4 system and the muscle-specific driver BG57-Gal4^14,15^. The effect of DVAP-V260I transgenic expression was compared and contrasted to that of two other transgenes (DVAP-WT1 and DVAP-WT2), which express different levels of the wild-type DVAP protein^16^. More specifically, the increase in DVAP immunoreactivity is 2.2-fold higher than in controls for the DVAP-WT2 line while DVAP-V260I and DVAP-WT1 exhibit comparable and lower levels of the same signal^16^.

Nuclear alterations have been associated with ageing and several neurodegenerative diseases including Parkinson's disease^17,18^. To assess whether our fly model for ALS8 exhibits changes in nuclear architecture, position and size, we stained nuclei within striated muscles of appropriate genotypes with a nuclear marker and the anti-lamin antibody^19-22^, which visualizes the nuclear envelope. To highlight the muscles, a DVAP-specific antibody was also added to the same samples (**Figure 1**). Confocal images were collected and detailed morphometric analyses were performed using an image analysis software. In control muscles, nuclei were found to be evenly distributed along the muscle fibers while in DVAP-V260I and DVAP-WT expressing muscles, nuclei exhibit a tendency to redistribute in closely associated clusters (**Figure 1**).

We conducted a nearest neighbor analysis to perform a quantitative evaluation of the distribution of nuclei along the muscle fibers of every genotype. A nearest neighbor analysis first identifies the closest neighbor for every nucleus by measuring the distance between the center of a given nucleus and the center of every other surrounding nucleus. This procedure is then repeated for every other nuclei along the muscle fiber. Finally, the shortest distance between nuclei within a specific muscle, is calculated by averaging the shortest distances of every nucleus and its nearest neighbors. (**Figure 2A**-**C**). Compared to controls, muscles expressing either the DVAP-V260I transgene or any of the transgenes overexpressing the wild-type protein, present a dramatic reduction in the average shortest distance between nuclei and, as a consequence, nuclei appear to be closely associated in clusters. The effect of the ALS causing allele DVAP-V260I is more severe than that associated with the overexpression of the wild-type protein, even if the strongest DVAP-WT2 transgene is used (**Figure 1** and **Figure 2D**).

Overexpression of either DVAP-V260I or DVAP-WT transgenes also exhibits a severe deterioration of nuclear architecture resulting in deformed nuclei with an elongated structure (**Figure 1**). This structural aberration was quantified by using the ImageJ software in which circularity is defined by the formula C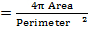, which measure the width to length ratio of every nucleus with C = 1 representing a perfect circle and C = 0 an infinitely elongated polygon. In control nuclei exhibiting a distinct round shape, C is equal to 1 while in the transgenic mutants a change in shape with consequent loss of circularity, causes a significant deviation from this value (**Figure 1** and **Figure 3**).

We also found that in muscles expressing the same transgenes, nuclei display a marked enlarged nuclear volume compared to controls, although the ALS causing allele appears to be more efficient in inducing this phenotype compared to the DVAP-WT transgenes (**Figure 4**).

Nearly all neurodegenerative diseases are characterized by the intracellular accumulation of aggregates containing the pathogenic protein. We made 3D reconstructions and volume renderings of nuclei and we found that in muscles expressing the mutant transgene or overexpressing the wild-type protein, DVAP immuno-reactivity formed clusters and that some of them were also localized into the nuclei (**Figure 5**). Conversely, in control NMJs, DVAP immuno-reactivity is faintly dispersed throughout the muscle fiber and is excluded from the nucleus^16^.


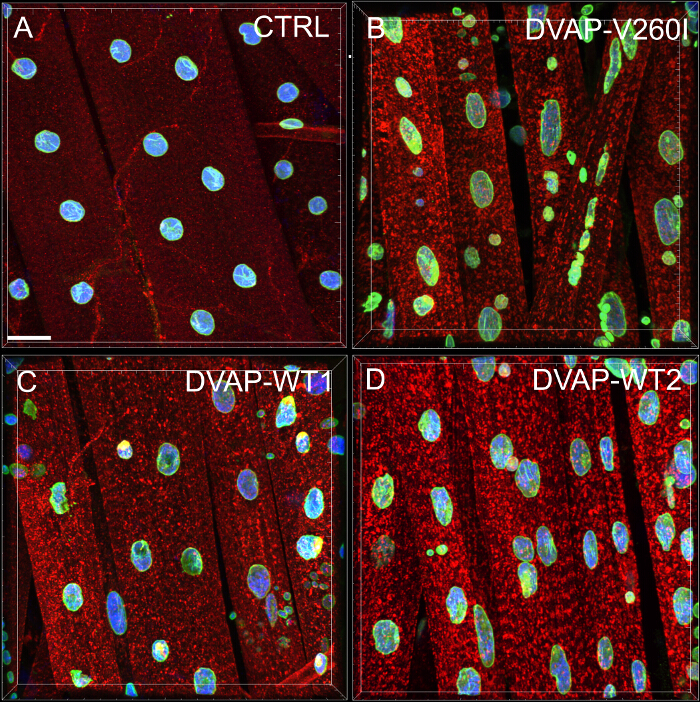
**Figure 1: Confocal images of myonuclei within striated muscles expressing either the DVAP-WT or the DVAP-260I transgenes. **(**A**) BG57-Gal4/+ control, (**B**) BG57;DVAP-V260I, (**C**) BG57;DVAP-WT1 and (**D**) BG57;DVAP-WT2 muscles expressing the indicated transgenes are stained with antibodies specific for DVAP (red signal), lamin (green signal) and with a nuclear specific marker to visualize the nuclei (blue signal). Scale bar = 30 µm Please click here to view a larger version of this figure.


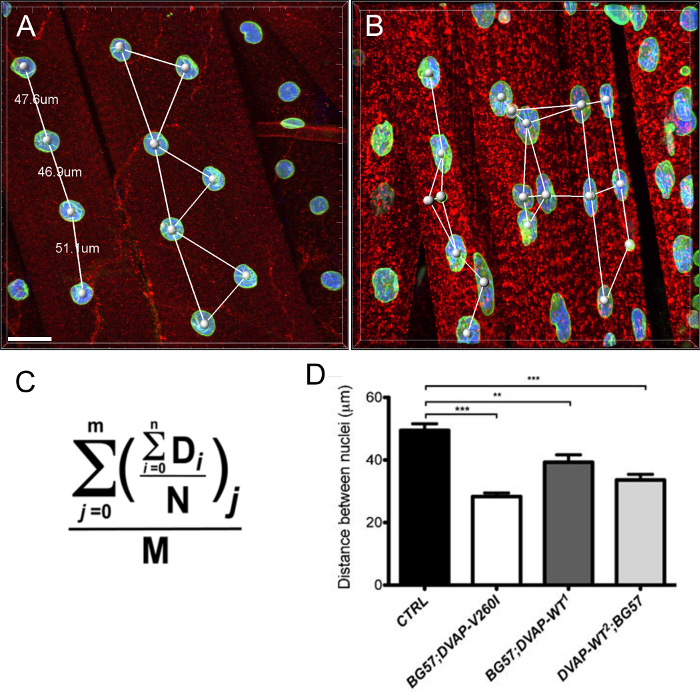
**Figure 2: Nearest neighbor analysis to determine the average distance between a nucleus and its single closest neighbor. **(**B**) Representative results showing altered nuclear positioning in muscles overexpressing the DVAP-WT2 transgene when compared to controls in (**A**). Average nuclear distance in muscles of the indicated genotypes was estimated using the formula in (**C**) and the data are reported in (**D**). Larval NMJs are stained with antibodies specific for DVAP (red signal), lamin (green) and with a nuclear marker (blue signal). Asterisks denote statistical significance. ***P <0.001, **P <0.01. For the statistical analysis of this experiment and all the experiments reported below a one-way ANOVA test was used and a Tukey's multiple comparison test was applied as a post-hoc test when differences between genotypes were found to be significant by the ANOVA test. Error bars represent SEM. Scale bar = 30 µm Please click here to view a larger version of this figure.


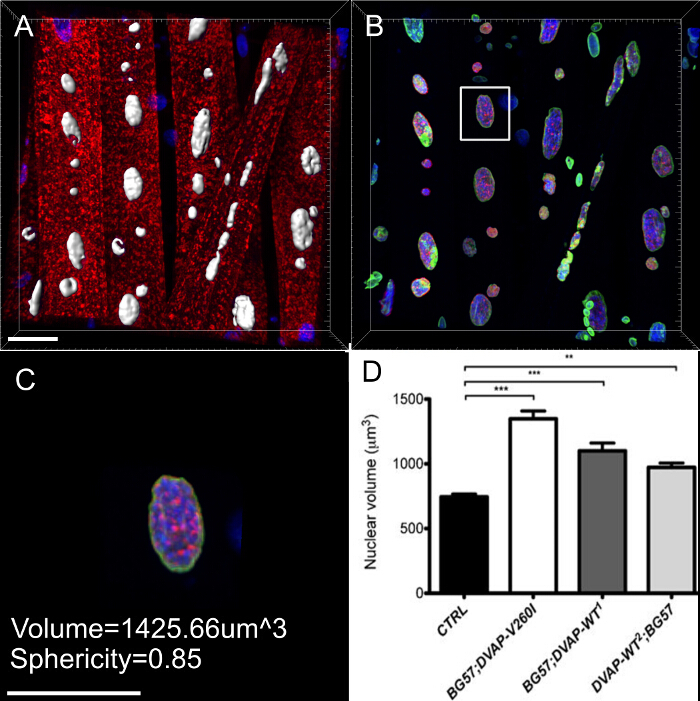
**Figure 3: Images showing representative steps in the calculation of the nuclear volume. **(**A**) A representative image showing segmented nuclei using the surface creation wizard. Nuclei at the border of the images were ignored. (**B**) Image showing the nuclear DVAP signal after the surrounding DVAP staining has been masked by using the surface created in the nuclear marker channel. (**C**) Surface layer provides information of additional parameters including the nuclear volume and the sphericity. (**D**) Data on nuclear volume of various genotypes. Asterisks denote statistical significance. Dissected NMJs were stained with anti-DVAP antibodies (red signal), anti-lamin antibodies (green signal) and a nuclear marker (blue signal). ***P <0.001, **P <0.01. Error bars represent SEM. Scale bar = 30 µm Please click here to view a larger version of this figure. 


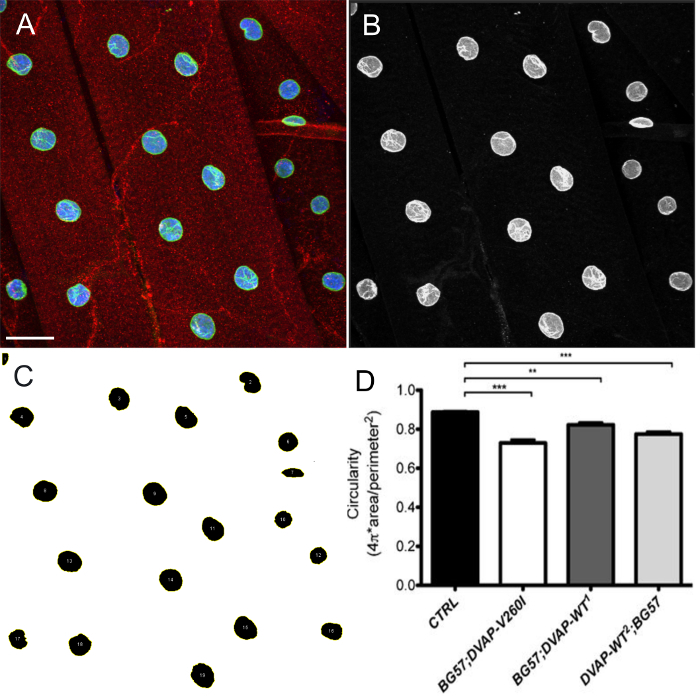
**Figure 4: Images showing representative steps in the estimation of nuclear shape by ImageJ.  **Maximum intensity projection of images were analyzed using ImageJ to estimate circularity of nuclei within muscles. (**A**) A representative example of the intensity projection of the three channel image as in step 7.9 of the protocol. (**B**) Image showing step 7.10 of the protocol in which channels are split and the nuclear marker channel is selected.****(**C**) A representative image showing that after applying intensity threshold to segment the nuclei and ROI manager plugin in ImageJ, all the nuclei of interest can be selected and their shape measured through Shape descriptors (steps 7.11-7.15). (**D**) Quantification of the circularity of various genotypes. On the larval NMJs, the red signal indicates DVAP staining while the green outlines nuclei and corresponds to the lamin staining. The interior of every nucleus is labelled in blue due to the staining with a nuclear marker. Asterisks denote statistical significance. ***P <0.001, **P <0.01. Error bars represent SEM. Scale bar = 30 µm Please click here to view a larger version of this figure.


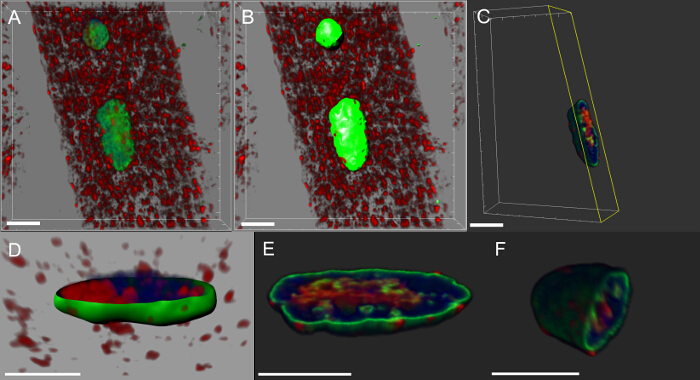
**Figure 5: Images showing specific steps in the creation of volume renderings of myonuclei**. (**A**) Image showing 3D intensity blended view of muscles stained with DVAP protein (red), the lamin (green) and the DNA marker (blue). (**B**) Image representing a surface layer generated by using the lamin channel to segment the nuclei. (**C**) Image representing a nucleus in which the surface layer has been used to mask the DVAP signal outside the selected nucleus. Highlighted in yellow is a clipping plane that has been added to the image. Its angle of view and position can be interactively adjusted to visualize the distribution of signal inside the nucleus. (**D**) An image reporting a cross-sectional view of the nuclear surface layer created using the lamin channel merged with unmasked DVAP and nuclear marker signals. (**E** and **F**) Additional sectioned volume renderings of the same nucleus. Scale bar = 10 µm Please click here to view a larger version of this figure.

## Discussion

In the past, morphological variations within and among experimental groups were rarely taken into account. However, the application of quantitative methods is now becoming the norm in comparative studies of morphology and mathematical description of anatomical forms are calculated. The use of quantitative analyses in assessing the effects of genetic manipulations on specific cellular processes, hold promise in enhancing our ability to detect morphological changes and in improving the accuracy with which these changes are described. Furthermore, statistical analysis of quantitative data allows us to evaluate whether observed differences between phenotypes are significant.

In striated muscles, nuclei exhibit a distinct rounded structure and are evenly distributed along the muscle fiber. Although the molecular mechanisms establishing and maintaining size, shape and architecture of nuclei are not known, these nuclear features are likely to play a fundamental role in controlling muscle function. Indeed, several myopathies are caused by mutations in genes regulating morphology and position of nuclei within muscles. The functional importance of shape and distribution of nuclei within a cell is not limited to muscles. Accumulating evidence shows that nuclear defects are also associated with neurodegenerative diseases such as Parkinson's disease^18,24^. Additionally, we are beginning to appreciate that morphology, size and intracellular distribution of other organelles including endoplasmic reticulum and mitochondria may have functional consequences. For instance, alterations in mitochondrial morphology are associated with neurological disorders such as optic atrophy type-1 (OPA1) and Charcot-Marie-Tooth type 2A neuropathy^25^.

To assist in the process of elucidating the molecular mechanisms underlying these important processes, we propose to combine high resolution confocal data with imaging software and morphometric analyses to quantitatively evaluate how genetic manipulations can affect shape, size, and location of nuclei within muscle fibers. The power and versatility of *Drosophila* genetics together with the highly stereotyped nature of the neuromuscular system in *Drosophila* larvae make the larval NMJ an experimental model particularly suitable for this type of analyses. At the larval NMJs, phenotypic analysis can be performed at a single synapse resolution allowing an accurate morphometric analysis where a number of NMJs can be studied within the same fly and even the same identifiable NMJ can be compared between flies of different genotypes^3,4^.

Phenotypic characterization of nuclear position, shape and size at the *Drosophila* larval NMJ starts by performing immunostaining of dissected NMJs with antibodies that highlight muscles and nuclei within muscles. In the protocol outlined in this paper, myonuclei were stained with polyclonal antibodies against lamin, a marker of the nuclear envelope, with a nuclear marker highlighting the nuclear interior and with antibodies specific to DVAP to stain the whole muscle. The lamin antibodies used in these experiments were kindly provided by Paul Fisher^19-22^ but alternative sources of anti-lamin antibodies can be used. Additionally, a number of other antibodies specific for the nuclear envelope are commercially available. Finally, nuclear markers, such as DAPI and propidium iodide, are also available while muscles could be visualized by staining with anti-actin or anti-tubulin antibodies. If antibodies other than those used in this experimental procedure are employed, the immunostaining protocol will require extra-steps in which fixation conditions and working concentrations for the new antibodies will need to be optimized. One critical step in this protocol, especially when volume renderings need to be analyzed, is the mounting of the samples on the slide. In this case, it is important to include spacers between the slide and the coverslip so that the specimens do not get squashed. Three bands of cellulose tape wrapped around the slide on both sides of the coverslip represent an easy way of making spacers.

While ImageJ was used for 2D images, most of the 3D multi-channel image analyses presented in this paper, were done by using Imaris because of its in-house availability. However, any other similar commercial software package can be used for these applications.

There are several open-source (for instance, ImageJ, CellProfiler, Vaa3D, Icy, KNIME and others) and commercial software platforms available for the analysis of confocal images. ImageJ^26^, the free software from the NIH or its more enhanced version, known as FiJi^27^, has a large number of import filters, macros and plugins available for the worldwide imaging community. Most of these plugins are focused on processing the information on a slice-by- slice manner. There are also plugins available for the visualization and analysis of multichannel 3D images. However, they are often designed for a specific task and users may need to extend or adapt these plugins to their own needs. On the other hand, commercial platforms target relatively inexperienced users and are often focused on ease-to-use, broad coverage of image-processing tasks with incredible speed.

The experimental procedure together with the quantitative phenotypic analysis outlined in this protocol, can assist in elucidating the molecular mechanisms controlling organelle morphology and their distribution within a cell. However, this approach has the obvious limitation of analyzing these processes at a specific end-point. The process of controlling morphology and distribution of organelles is likely to be very dynamic and to vary not only between different cell types but also within the same cell depending on the developmental or physiological status. A further implementation of this analysis would be represented by time lapse imaging that permits changes in organelle morphology and position to be monitored over time.

## Disclosures

The authors have nothing to disclose.
